# Gut Microbiome Signature Are Correlated With Bone Mineral Density Alterations in the Chinese Elders

**DOI:** 10.3389/fcimb.2022.827575

**Published:** 2022-03-31

**Authors:** Yangyang Wang, Xiaoguang Gao, Jing Lv, Yuhong Zeng, Qingmei Li, Liping Wang, Yuanyuan Zhang, Wenjie Gao, Jihan Wang

**Affiliations:** ^1^ School of Electronics and Information, Northwestern Polytechnical University, Xi’an, China; ^2^ Clinical Laboratory of Honghui Hospital, Xi’an Jiaotong University, Xi’an, China; ^3^ Department of Osteoporosis, Honghui Hospital, Xi’an Jiaotong University, Xi’an, China; ^4^ Department of Cardiology, Honghui Hospital, Xi’an Jiaotong University, Xi’an, China; ^5^ Department of Spine Surgery, Sun Yat-Sen Memorial Hospital, Sun Yat-Sen University, Guangzhou, China; ^6^ Xi’an Key Laboratory of Stem Cell and Regenerative Medicine, Institute of Medical Research, Northwestern Polytechnical University, Xi’an, China

**Keywords:** osteoporosis, postmenopausal osteoporosis, bone mineral density, gut microbiome, metagenomics

## Abstract

**Objective:**

Osteoporosis (OP), clinically featured with a low bone mineral density (BMD) and high risk of bone fracture, has become a major risk factor of disability and death in the elders, especially in postmenopausal women. The gut microbiome (GM) is thought to be implicated in bone metabolism. Herein, we clarified the composition signature and gene functional profile of GM in older people with normal and low BMD.

**Design and Methods:**

A total of 455 participants underwent the BMD measurement and biochemical detection. GM analysis was further performed on 113 cases of postmenopausal women and men aged over 50, including both 16S rRNA and metagenomic sequencing.

**Results:**

Generally, the BMD value was significantly lower in the older age groups, especially in the postmenopausal women. Consistently, we observed obvious vitamin D deficiency or insufficiency in females (compared to the male, *P* < 0.0001). The results from 16S rRNA sequencing revealed higher numbers of OTUs and diversity indexes in females than in males. The abundance in composition of *Firmicutes* and *Clostridiales* were correlated with the BMD values in females. LEfSe analysis discovered several enriched bacteria taxons in OP and normal control (NC) subgroups. A positive correlation between the number of genes and BMD values was observed in females based on metagenomic sequencing analysis. Furthermore, we identified the connecting modules among the GM composition – gene functional signature – BMD value/T score in both females and males.

**Conclusions:**

This study provides evidences upon which to understand the mechanisms of the effects of GM on bone health, consequently revealing the physiology status and potential diagnostic/therapeutic targets based on GM for OP and postmenopausal osteoporosis (PMOP). Besides, the status of vitamin D deficiency or insufficiency need to be concerned and improved in the Chinese people.

## Introduction

Osteoporosis (OP) is the most common bone disease, characterized by low bone mass and damaged bone tissue microstructure, leading to increased bone fragility that is prone to fracture (Greenwood et al., 2018). OP can occur at any age, but it is more common in postmenopausal women (postmenopausal osteoporosis, PMOP) and older men (older than 50 years) (Qaseem et al., 2017). Osteoporotic fracture (OF) caused by OP is one of the main risk factors of disability and death in the elders (Si et al., 2015; Compston et al., 2019). Women’s risk of developing OFs during their lifetimes (40%) is greater than the sum of the risks for ovarian cancer, breast cancer, and endometrial cancer. For men, the risk of developing OFs (13%) is higher than that of prostate cancer (Lin et al., 2015).

Bone metabolism is maintained through a combination of several regulated host mechanisms, including mineral absorption, hormonal control, and immunomodulation (Hansen et al., 2015; Weitzmann and Ofotokun, 2016; Fan et al., 2017). Recently, studies have demonstrated gut microbiome (GM) involving in the regulation of bone homeostasis and the mechanism of osteoporosis (Quach and Britton, 2017; D'Amelio and Sassi, 2018; Cooney et al., 2020; Ding et al., 2020; He et al., 2020). The gut microbiome is the entire complement of commensal, symbiotic, and pathogenic microorganisms living in our intestines, and the development of effective sequencing technologies has made it possible to study the composition of the GM, while identifying its importance for both health and disease, including OP (Xu et al., 2017; Biver et al., 2019; Schepper et al., 2020). The potential mechanisms underlying how GM affects bone metabolism include their influence on nutrient absorption and the intestinal mucosal barrier, as well as their influence on the immune system and the gut – brain axis, and so forth (Chen et al., 2017). Studies have also suggested that the GM may furnish effective novel biomarkers in the diagnosis/prognosis of bone disease and other phenotypic traits (Biver et al., 2019). Current research, although limited, revealed the linkages between GM and bone metabolism (Luo et al., 2015; Aurigemma et al., 2018); therefore, further exploration of this relationship is a promising area for bone health and OP research. Most existing studies investigated the mechanism of GM on bone health using animal models (Yan et al., 2016; Liu et al., 2019). However, more studies with clinical samples (Wang et al., 2017; Das et al., 2019) are still needed to explore the correlations between GM and BMD changes in OP.

In this study, we collected fecal samples from the elder subjects (including postmenopausal women and men aged 50 years and above) with OP, osteopenia (ON), or a normal bone mass (normal control, NC), and performed a comprehensive analysis of the GM composition as well as GM functional profiles using 16S rRNA combining metagenomics sequencing. The results may provide a foundation for understanding the potential mechanisms of the effects of GM on bone health, consequently revealing the possible diagnostic GM biomarkers for this widespread disease.

## Material And Methods

### Study Design and Population

**Figure 1:**
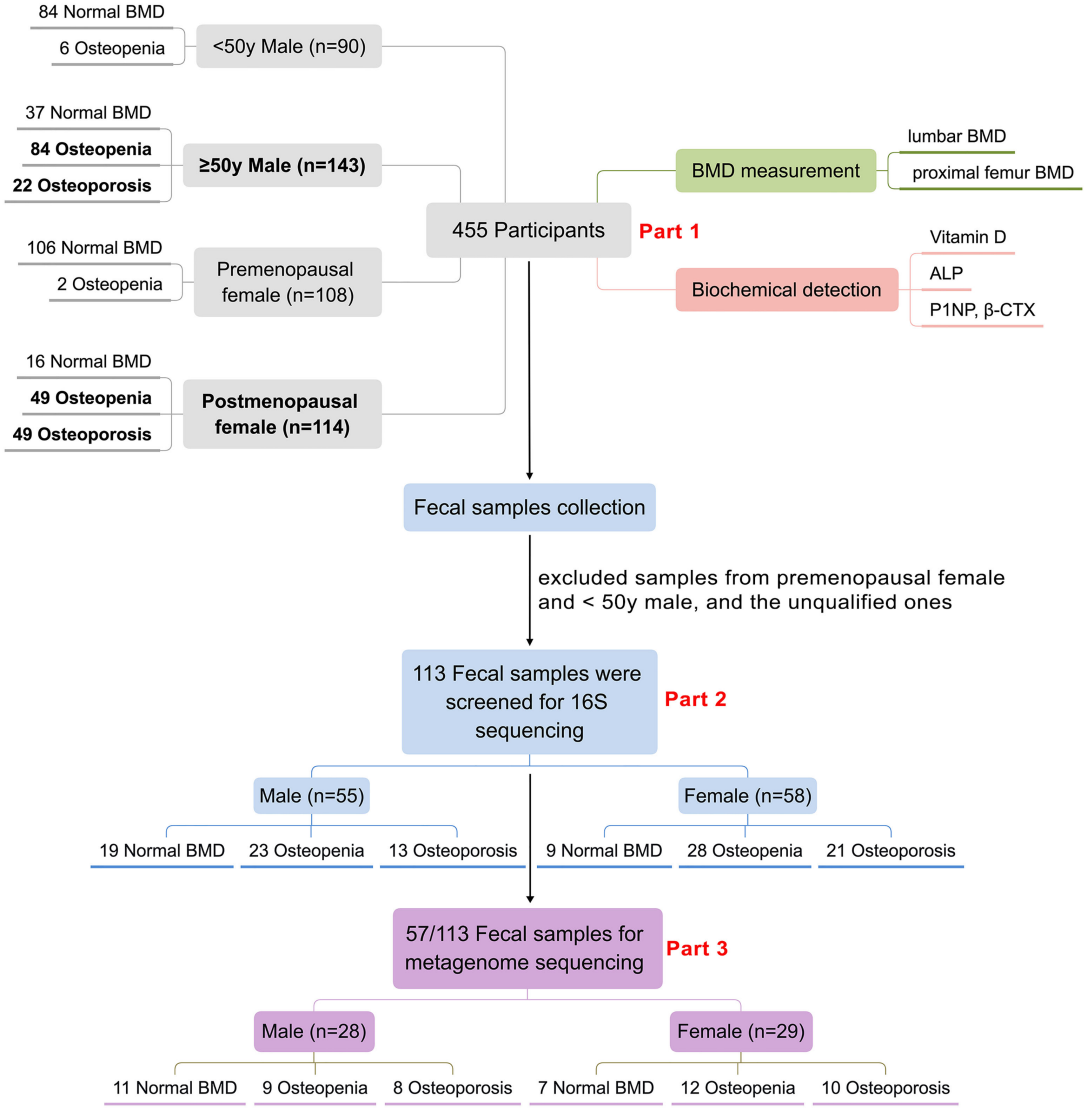
Demographics of the study population. 455 participants were involved in this study, biochemical detection including serum vitamin D, ALP, P1NP and CTX as well as DXA-BMD measurement of the 455 participants were analyzed (Part 1). 113 participants including 55 males and 58 females were screened for GM 16S sequencing and analyses (Part 2), further 57 samples from the above 113 samples were selected for GM metagenome sequencing and analyses (Part 3).

In 2018, China launched the first epidemiological survey of OP in Chinese residents, organized by the National Health Commission of China. The BMD measurements and laboratory testing took place at Honghui Hospital, Xi’an Jiaotong University, for residents near Xi’an (Xi’an, Shaanxi, China). Residents or patients with any malignancy, chronic liver disease, heart disease, kidney disease, diabetes, or disease related with secondary OP (hyperthyroidism, steroid abuse, Cushing’s syndrome, hyperparathyroidism, *etc.*) were excluded. In addition, BMD measurements were not available in the following situations: (1) during pregnancy; (2) those orally administered with drugs within 2–6 days of measurement that affect image development; (3) when radioisotope inspections were carried out within 3 days; (4) those who cannot lie on the examination bed, or cannot adhere to the 5-min test; (5) those with a severe deformity of the spine or metal implants on the spine. Finally, a total of 455 residents participated in BMD examination and laboratory biochemical testing as a part of the OP epidemic survey. We then collected the fecal samples from the participants for GM analysis. After exclusion of unqualified fecal samples such as samples less than 1 g or samples from younger participants (including males < 50 years and premenopausal females), 113 subjects [including 58 postmenopausal women and 55 men (≥50 years old)] were finally selected for further GM research. The GM analysis mainly contained two parts. Firstly, all of the 113 fecal samples were included for 16S ribosomal RNA sequencing. Then, parts of the 113 fecal samples were chosen for further metagenome sequencing. Specifically, we tried to ensure that there was little difference in the sample size and the basic information (age, BMI) between female and male, and among the three subgroups in both female and male for the metagenome analysis. Finally, 57 fecal samples (including 29 females and 28 males) from the previous 113 samples were selected for metagenome sequencing. **Figure 1** summarizes the demographics of the study design and population.

### BMD Measurements

BMD measurements of the lumbar vertebrae (L_1-4_) and proximal femur for 455 participants were performed using a standard dual-energy X-ray absorptiometer (Discovery Wi, Hologic, Marlborough, MA, USA). We collected information pertaining to the subjects’ BMD values (g/cm^2^), T-scores, and Z-scores. The T score and Z score reference ranges were calculated with data from healthy Asian population provided by the bone densitometry equipment manufacturer. According to the diagnostic criteria for OP in postmenopausal women and men over 50 years old, participants with T-scores ≤ –2.5 at any site were diagnosed as having OP (when accompanied with one or more fractures, defined as severe OP), –2.5 < T-scores < –1.0 as having ON, and T-scores ≥ –1.0 for normal bone mass. In this study, none of the participants had a history of fractures.

### Biochemical Detection

Fasting venous blood samples were collected from the 455 participants for biochemical testing. Serum 25-hydroxyvitamin D (vitamin D), alkaline phosphatase (ALP), procollagen type 1 N-peptide (P1NP), and C-terminal cross-linking telopeptide of type 1 collagen (CTX) were detected using the Roche electrochemiluminescence system.

### Fecal Sample Collection and DNA Extraction

We screened a total of 113 fecal samples from the participants for GM analyses. Detailed information involving the fecal sample collection and DNA extraction refers to our previous study (Wang et al., 2017). Briefly, the bacterial genome in each sample was extracted using QIAamp Fast DNA Stool Mini Kit (Qiagen, Hilden, Germany) according to the manufacturer’s instructions.

### 16S rRNA Polymerase Chain Reaction (PCR), Library Preparation, and Illumina Sequencing

Detailed information involving 16S rRNA PCR and Illumina sequencing refers to our previous study (Wang et al., 2017). Briefly, the bacterial 16S ribosomal RNA gene V3-V4 region was amplified by using the TransGen AP221-02 Kit (TransGen, Beijing, China), with the PCR primers as follows: 338F 5’-ACTCCTACGGGAGGCAGCAG-3’ and 806R 5’- GGACTACHVGGGTWTCTAAT-3’. The amplicon samples were extracted, purified and quantified by using the AxyPrep DNA Gel Extraction Kit (Axygen Biosciences, CA, USA) and QuantiFluor™-ST (Promega, Madison, WI, USA). Finally, purified amplicons were paired-end sequenced (2 × 250 bp) on an Illumina NovaSeq platform (Illumina, San Diego, CA, USA).

### Data Processing and Analysis for 16S

The Uparse software (Uparse v7.0.1001) (Edgar, 2013) and Quantitative Insights Into Microbial Ecology (QIIME) software (Kuczynski et al., 2012) were utilized for 16S rRNA sequences analysis, and sequences with ≥ 97% similarity were assigned to the same operational taxonomic unit (OTUs). We further selected a representative sequence for each OTU and annotated the taxonomic information for the representative sequences based on the RDP classifier (Cole et al., 2014). The QIIME calculated both alpha- (within samples) and beta- (among samples) diversity. The indexes including Shannon, Simpson, Chao1, and ACE were analyzed as alpha diversity, the Shannon and Simpson indexes indicate the community diversity, the Chao1 and ACE indicators reflect the community richness. We used both weighted and unweighted unifrac for Principal Coordinate Analysis (PCoA) to get principal coordinates in this study.

### Library Construction and Sequencing for the Metagenome

A total amount of 1 μg of DNA per sample was used as the input material for the DNA sample preparations. Sequencing libraries were generated using the NEBNext^®^ Ultra™ DNA Library Prep Kit for Illumina (NEB, USA), according to the manufacturer’s recommendations, and index codes were added to attribute sequences to each sample. Briefly, the DNA sample was fragmented by sonication to a size of 350 bp, and then the DNA fragments were end-polished, A-tailed, and ligated with the full-length adaptor for Illumina sequencing with further PCR amplification. Finally, the PCR products were purified (AMPure XP system), and libraries were analyzed for size distribution by an Agilent2100 Bioanalyzer and quantified using real-time PCR. The clustering of the index-coded samples was performed on a cBot Cluster Generation System, according to the manufacturer’s instructions. After cluster generation, the library preparations were sequenced on an Illumina HiSeq platform, and paired-end reads were generated. The above procedures were implemented at the Novogene Bioinformatics Technology Co., Ltd.

### Gene Prediction and Abundance Analysis

The reads assembly was conducted by using SOAP denovo (Version 2.04). Both the single and assembled Scaftigs (≥ 500 bp) predicted the ORF by MetaGeneMark V2.10 software (Tang et al., 2013), and the length information shorter than 100 nt was filtered from the predicted result with default parameters. For the predicted ORF, CD-HIT V4.5.8 software (Fu et al., 2012) was adopted to obtain the unique initial gene catalogue (the genes here refer to the nucleotide sequences coded by unique and continuous genes). The clean data of each sample was mapped to the initial gene catalogue using Bowtie2.2.4 and to get the number of reads (Langmead and Salzberg, 2012). The genes with the number of reads ≤ 2 in each sample were filtered to obtain the gene catalogue (Unigenes) eventually used for subsequent analysis. Based on the number of mapped reads and the length of the gene, the abundance information of each gene in each sample was summarized.

### Kyoto Encyclopedia of Genes and Genomes Functional Database Annotations

DIAMOND software (V0.9.9) was adopted to blast Unigenes to the KEGG database (Liu et al., 2020). For each sequence’s BLAST result, the best BLAST hit was used for subsequent analysis. The relative abundances of different functional hierarchies were calculated. The relative abundance of each functional hierarchy was equal to the sum of all relative abundances annotated to that functional level. Based on the function annotation result and the gene abundance table, the gene number table of each sample in each taxonomy hierarchy was obtained. The number of genes with a particular function in a sample was equal to the number of genes that annotated to this function, and the abundance could not be zero.

### Statistical Analysis

Statistical analysis and figures generation were performed using the R 3.6.0 tool, GraphPad Prism 5.01, and Medcalc 19.0.4 softwares. The Wilcoxon rank-sum test was performed to analysis differences of the clinical variables, gut microbiome composition, and KEGG functional enrichment between different groups, with *P* < 0.05 indicating statistical differences. The “PerformanceAnalytics” package in R was applied for basic information correlation analysis. Pearson correlation test in R was used for correlation analysis between GM composition and BMD values, as well as between KEGG functional annotation and BMD values. Linear discriminant analysis (LDA) combined with effect-size measurements (LEfSe) analysis was utilized to identify the relatively enriched bacteria taxon in each group or subgroup, with *P* < 0.05 and LDA scores > 4 indicating significant enrichment. MedCalc software was utilized for the receiver operating characteristic (ROC) analysis to evaluate the performance of candidate classifiers in the differential diagnosis between the disease (the OP group) and non-disease (non-OP, including both NC and ON groups) groups, the level of significance was set at *P* < 0.05.

## Results

### Participant Characteristics Analyses

**Figure 2:**
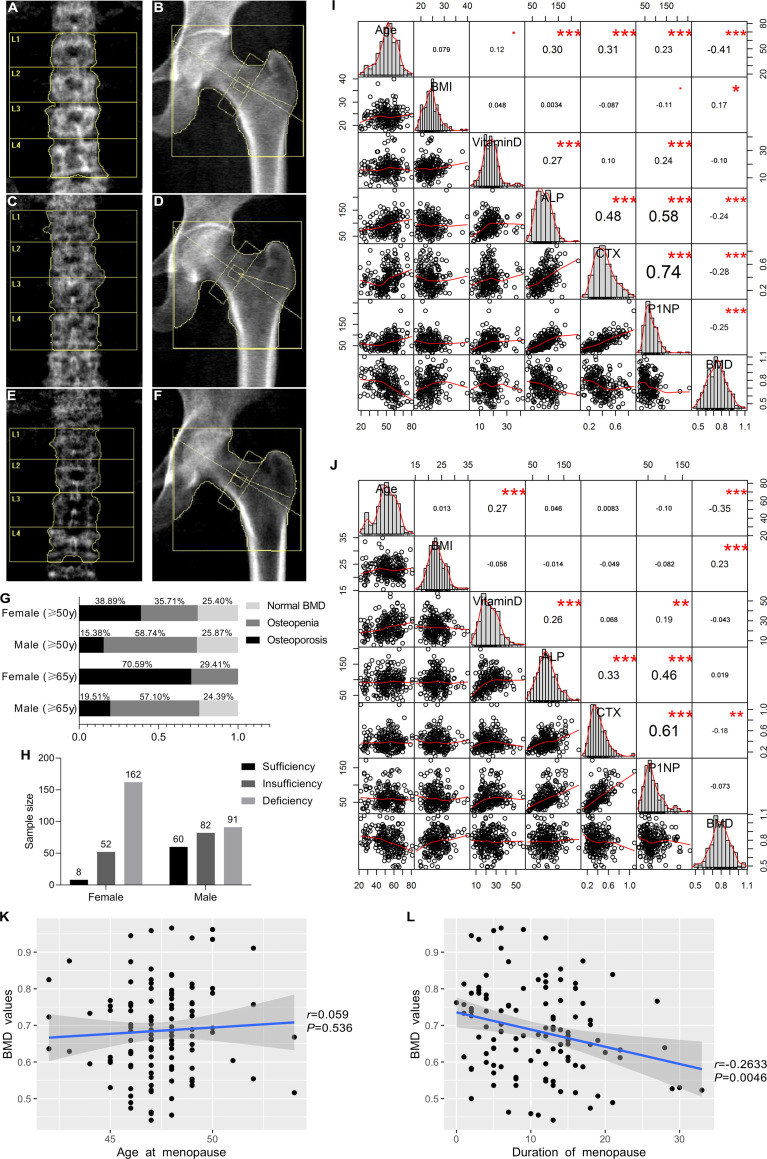
DXA scan images of the BMD measurement and epidemiological investigation as well as correlation analysis of clinicopathological characteristics and BMD values. Lumbar spine (L_1–4_) and total hip images in normal BMD group **(A, B)**, osteopenia (ON) group **(C, D)**, and osteoporosis (OP) group **(E, F)**. **(G)** Epidemiological investigation of OP and ON in females and males in our study. **(H)** Vitamin D deficiency and insufficiency proportions. **(I, J)** Correlation analysis between clinicopathological characteristics and BMD values for female subjects and male subjects, respectively. This image presents bar graphs that show the distribution of variables as well as the scatter plots for two variables; the numbers in the image indicate the correlation coefficient of the two variables, while red dots and asterisks indicate the degree of statistical significance, •0.05 < *P* < 0.1, *P < 0.05, **P < 0.01, ***P < 0.001. **(K)** Correlation between the menopausal age and BMD values in the females. **(L)** Correlation between the duration of menopause and BMD values in the females.

In this study, all the 455 participants were involved for both BMD detection and biochemical testing. **Figures 2A–F** showed the scan images obtained from the DXA detector. For premenopausal women and men under 50 years old, only a few participants had ON (1.85% for females and 6.67% for males), while the BMD value was significantly lower in the older age groups, with aged females were accompanied by more severe BMD loss than males. More than a third of females over 50 years old had OP, and the prevalence was more significant in people older than 65 years old. For males older than 50 years old, more than half had ON (**Figure 2G**). The serum concentrations of vitamin D, ALP, CTX, and P1NP were detected. Generally, vitamin D deficiency is defined as a serum 25-hydroxyvitamin D level of less than 20 ng/mL, and insufficiency is defined as a serum 25-hydroxyvitamin D level of 20–30 ng/mL. In this study, obvious vitamin D deficiency as well as insufficiency were observed, especially in female participants, in whom only 3.60% were shown to have sufficient vitamin D levels (**Figure 2H**). The correlation between age, body mass index (BMI), biochemical detection and the BMD values were analyzed in this study. The BMI values were positively correlated with the BMD values, while age was negatively correlated (**Figures 2I, J**). The serum concentrations of ALP, CTX, and P1NP were all higher in the postmenopausal women than in the premenopausal females and males (**Table 1**). They were also positively and reciprocally correlated with each other for both the female and male participants (**Figures 2I, J**). In postmenopausal women, the age of menopause had no obvious correlation with the BMD values (**Figure 2K**), while the duration of menopause was negatively related with the BMD values (**Figure 2L**).

**Table 1:** Characteristics of the 455 participants in this study.

		n	Age	M-age	BMI	Vitaminng/mL	ALPU/L	CTXng/mL	P1NPng/mL	BMDg/cm^2^	T score
Premenopausal female		108	41.33 ± 8.97		24.32 ± 3.57	16.26 ± 5.51	82.06 ± 26.36	0.30 ± 0.14	55.22 ± 27.82	0.80 ± 0.11	-0.91 ± 0.82
Postmenopausal female	NC	16	58.63 ± 4.21	47.13 ± 2.84	24.14 ± 3.26	17.96 ± 4.42	95.69 ± 28.35	0.45 ± 0.16	76.12 ± 27.72	0.86 ± 0.09	-0.56 ± 0.31
	ON	49	59.67 ± 6.46	46.94 ± 1.60	24.06 ± 3.18	17.98 ± 5.69	106.57 ± 32.69	0.46 ± 0.16	77.07 ± 26.44	0.73 ± 0.08^#^	-1.81 ± 0.38^#^
	OP	49	60.96 ± 6.72	47.73 ± 2.18	22.76 ± 2.74	18.22 ± 7.38	101.31 ± 34.13	0.44 ± 0.15	76.98 ± 37.09	0.59 ± 0.08^#△^	-3.10 ± 0.44^#△^
	Total	114	60.14 ± 6.70^*^	47.42 ± 2.08	23.51 ± 3.05	18.08 ± 6.29^* c^	102.78 ± 32.71^* a^	0.45 ± 0.16^* b^	76.89 ± 31.36^* c^	0.69 ± 0.12^* c^	-2.19 ± 0.98^* c^
< 50y Male		90	39.82 ± 8.36		23.28 ± 3.46	20.84 ± 7.85	91.53 ± 28.41	0.38 ± 0.14	64.83 ± 28.53	0.83 ± 0.10	-0.99 ± 0.87
≥ 50y Male	NC	37	60.14 ± 5.74		23.47 ± 2.81	26.28 ± 11.62	94.05 ± 29.28	0.34 ± 0.14	55.93 ± 20.85	0.87 ± 0.08	-0.42 ± 0.57
	ON	84	59.85 ± 7.95		23.00 ± 2.75	26.02 ± 9.02	94.63 ± 28.38	0.42 ± 0.15^#^	66.04 ± 24.68^#^	0.74 ± 0.07^#^	-1.77 ± 0.39^#^
	OP	22	61.05 ± 5.85		21.17 ± 2.82	23.05 ± 7.44	99.59 ± 32.93	0.45 ± 0.19^#^	65.66 ± 32.49	0.59 ± 0.07^#△^	-2.99 ± 0.51^#△^
	Total	143	60.10 ± 7.11^*^		22.84 ± 2.85	25.63 ± 9.55^*^	95.24 ± 29.19	0.40 ± 0.16	63.37 ± 25.33	0.75 ± 0.11^*^	-1.61 ± 0.94^*^

NC, normal control; ON, osteopenia; OP, osteoporosis; M-age, age at menopause; ALP, alkaline phosphatase; CTX, C-terminal cross-linking telopeptide of type 1 collagen; P1NP, procollagen type 1 N-peptide; BMD, bone mineral density. Compared with the younger group in the same gender; ^*^P < 0.05, compared with the NC subgroup in the same gender; ^#^P < 0.05, compared with the ON subgroup in the same gender; ^△^P < 0.05, total postmenopausal female compared with total ≥ 50y male; ^a^P < 0.05, ^b^P < 0.01, ^c^P < 0.0001.

### GM Diversity Was Altered Between Females and Males

**Figure 3:**
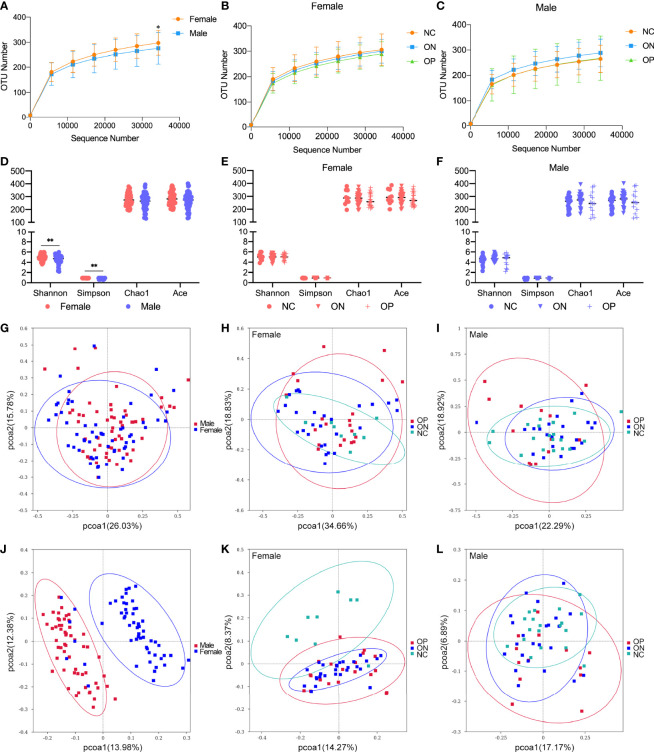
Observed OTUs, alpha diversity indexes, and OTU-level PCoA analysis in females and males. **(A–C)** Observed OTUs in different levels of sequences/sample in female and male subjects, and in the subgroups of females and males. **(D–F)** Alpha diversity indexes including Shannon, Simpson, Chao1, and ACE in female and male subjects, and in the subgroups of females and males. **(G–I)** OTUs-based weighted unifrac PCoA analysis of the 16S sequencing in female and male subjects, and in the subgroups of females and males. **(J–L)** OTUs-based unweighted unifrac PCoA analysis of the 16S sequencing in female and male subjects, and in the subgroups of females and males. *P < 0.05, **P < 0.01.

In this research, we focused on GM alterations in postmenopausal women and men over 50 years old, since there was no significant reduction of bone mass in younger adults. A total of 113 fecal samples (including from 58 females and 55 males) were chosen for 16S ribosomal RNA sequencing. There was no significant difference in age and BMI between the female and male groups or among the NC, ON, and OP subgroups (**Table S1**). Overall, the observed OTUs tended to be higher in females compared to males (**Figure 3A**). Specifically, the number of OTUs ranged from 208 to 393 (297.22 ± 49.47) for the female subjects and from 146 to 416 (275.64 ± 63.19) for the male subjects (**Table S2**). In both the female and male groups, there was no obvious difference of observed OTUs among the NC, ON, and OP subgroups (**Figures 3B, C**). In the females, number of OTUs was moderately decreased in the low-BMD groups compared to the NC subjects, which was consistent with our previous studies (Wang et al., 2017). Alpha diversity (including indexes of Shannon, Simpson, Chao1, and ACE) of bacterial communities showed a higher GM diversity in females than that from males (*P* < 0.01 for Shannon and Simpson indexes, **Figure 3D**), while the differences of alpha diversity indexes among NC, ON, and OP subgroups were not that significant (**Figures 3E, F**). The OTU level PCoA reflecting beta diversity revealed different beta diversity of microbiota signature between females and males, the female and male samples distribution in weighted unifrac PCoA (**Figure 3G**) was not that distinguishable as shown in unweighted unifrac method (**Figure 3J**), and the patterns exhibited fewer differences among the NC, ON, and OP subgroups in both female (**Figures 3H, K**) and male (**Figures 3I, L**).

Since the gender factor influences GM diversity, we analyzed GM composition and functional annotation in females and males separately in the following study.

### GM Landscape Were More Correlated With BMD Alterations in the Females Than the Males

**Figure 4:**
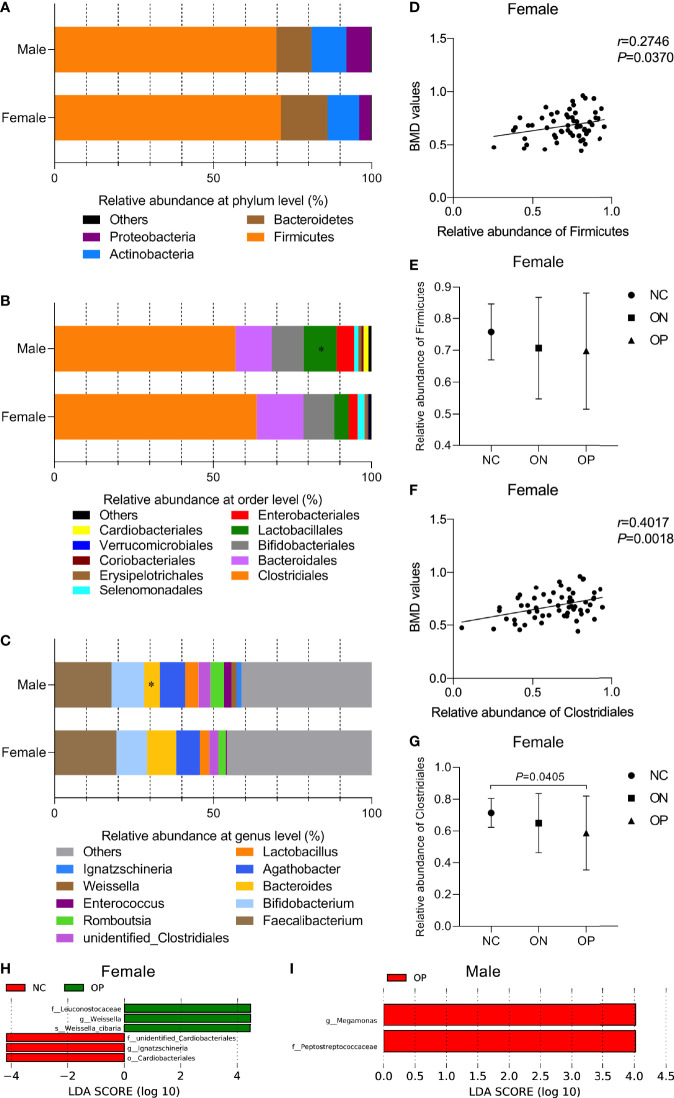
Relative abundance of GM composition and its correlation with BMD values at different taxonomic levels in females and males. **(A–C)** Relative abundance of GM composition in female and male groups at the phylum level, order level, genus level, respectively. Compared with the female group, *P < 0.05. **(D, E)** The correlation of phyla *Firmicutes* with BMD values and the relative abundance of *Firmicutes* in the three subgroups of females. **(F, G)** The correlation of order *Clostridiales* with BMD values and the relative abundance of *Clostridiales* in the three subgroups of females. **(H, I)** LEfSe analysis of enriched GM community in the three subgroups of females and males. *P* < 0.05, LDA value > 4.

To investigate the gut microbiota composition and further analyze whether the abundance of certain bacterial composition could be related to BMD changes in the participants, we performed Pearson correlation test between the relative abundances of bacterial species and BMD values at different taxonomic levels. At the phylum level (**Figure 4A**), *Firmicutes* dominated the microbiota (70.46% ± 16.87%), followed by *Bacteroidetes* (13.19% ± 12.93%), *Actinobacteria* (10.46% ± 12.84%), and *Proteobacteria* (5.53% ± 11.47%). Overall, there was no obvious difference in the relative abundance of the four phyla between females and males. In the female subjects, a positive correlation between the relative abundance of the phyla *Firmicutes* and the BMD values was observed (*r* = 0.2746, *P* < 0.05, **Figure 4D**), although there was no significant difference among the three subgroups (**Figure 4E**). At the order level (**Figure 4B**), *Clostridiales* (60.43% ± 21.34%), *Bacteroidales* (13.19% ± 12.93%), *Bifidobacteriales* (9.96% ± 12.7%), *Lactobacillales* (7.38% ± 14.66%), and *Enterobacteriales* (4.19% ± 8.76%) constituted more than 95.16% of the microbiota. Samples from males had a greater relative abundance of *Lactobacillales* than those from females (*P* < 0.05). The relative abundance of *Clostridiales* was positively correlated with the BMD values in the female subjects (*r* = 0.4017, *P* < 0.01, **Figure 4F**). With a decrease in the BMD, the relative abundance of *Clostridiales* was obviously reduced in the OP compared to the NC group (*P* < 0.05, **Figure 4G**). At the genus level (**Figure 4C**), *Faecalibacterium* (18.71% ± 14.28%), *Bifidobacterium* (9.96% ± 12.76%), *Agathobacter* (7.73% ± 10.07%), *Bacteroides* (7.09% ± 9.22%), and *Lactobacillus* (3.54% ± 10.12%) were the top five genera of the microbiota, and the relative abundance of *Bacteroides* was increased in the female compared to the male samples (*P* < 0.05). In the male group, we found no significant correlations between specific GM composition and the BMD values. For instance, the relative abundances of phyla *Firmicutes* and order *Clostridiales* were not that significantly correlated with the BMD values in male (*r_Firmicutes_* = -0.0138, *r_Clostridiales_* = 0.0275, *P* > 0.05, **Figures S1A, B**).

We then performed LEfSe to investigate the relatively enriched bacterial community in the three subgroups of females and males. We identified several taxons that were enriched in the NC and OP subgroups in females (*P* < 0.05, LDA scores > 4, **Figure 4H**). *Peptostreptococcaceae* (family) and *Megamonas* (genus) taxa were enriched in the OP subgroup (*P* < 0.05, LDA scores > 4), while no community in the NC or ON subgroups of males was enriched, as shown in **Figure 4I**. Overall, the variant enrichment of GM composition may assistance in distinguishing OP patients from NC subjects.

### Particular KEGG Functional Pathways Correlated With BMD Values in Females and Males

**Figure 5:**
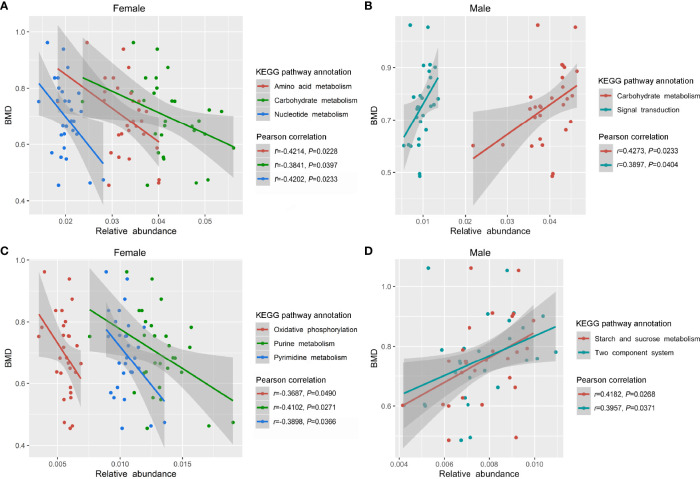
Relative enrichment of KEGG functional annotation and their correlation with BMD values in females and males. **(A)** Relative enrichment of carbohydrate metabolism, amino acid metabolism, nucleotide metabolism, and their correlation with BMD values in females. **(B)** Relative enrichment of carbohydrate metabolism, signal transduction, and their correlation with BMD values in males. **(C)** Relative enrichment of purine metabolism, pyrimidine metabolism, oxidative phosphorylation, and their correlation with BMD values in females. **(D)** Relative enrichment of two-component system, starch and sucrose metabolism, and their correlation with BMD values in males.

After the preliminary analysis of GM composition by 16S sequencing, we further conducted in-depth research at the genetic and functional levels by metagenomic sequencing. Specifically, 57 fecal samples (including 29 females and 28 males) from the previous 113 samples were selected for metagenome sequencing (**Table S3**). A range of 165,344 to 558,564 (401,527.30 ± 109,005.00) genes in the samples from females and 169,446 to 565,122 (377,208.30 ± 110,984.70) genes in the samples from males were captured, and the number of genes was slightly higher in the samples of female than that of male (**Figure S2A**). In the female group, we observed a positive correlation trend between GM gene numbers and the BMD values (*P* < 0.05, **Figures S2B, C**), while the correlation in males was not obvious (**Figure S2D**). The observed genes and their functions were annotated according to the KEGG database. Overall, the most abundant genes were in the metabolism category, followed by environmental information processing, genetic information processing, cellular processes, human diseases, and organismal systems (**Figure S3**). Generally, there was no clear difference in the profile of the KEGG functional pathways between the female and male subjects (**Figure S4**). We further observed that the relative enrichment of genes annotated with carbohydrate metabolism, amino acid metabolism, and nucleotide metabolism were all negatively correlated with the BMD values in female participants (*P* < 0.05, **Figure 5A**). With a decrease in the BMD, the relative enrichment of the three metabolism functional categories increased, but there was no statistical difference among the NC, ON and OP subgroup in females (**Figure S5A**). As for the male subjects, the relative enrichment of genes annotated with carbohydrate metabolism and signal transduction was positively related to the BMD values (*P* < 0.05, **Figure 5B**), and there were no significant differences among the NC, ON, and OP subgroups (**Figure S5B**). More specifically, the relative enrichment of purine metabolism, pyrimidine metabolism, and oxidative phosphorylation at level 3 of KEGG were negatively related to the BMD values in females (*P* < 0.05, **Figure 5C** and **Figure S5C**), and the relative enrichment of the two-component system as well as starch and sucrose metabolism were positively correlated with the BMD values in males (*P* < 0.05, **Figure 5D** and **Figure S5D**).

### Disease Status Identification With the Gene Functional Signature

Clinically, the T-score obtained from the dual-energy X-ray absorptiometer (DXA) measurements is still the globally accepted “gold-standard” method for the noninvasive diagnosis of OP; therefore, we could completely distinguish the disease group (OP) from the non-disease groups (non-OP, including both NC and ON groups) by the T-score in both females and males [area under curve (AUC) = 1, *P* < 0.001, **Table S4**]. The BTMs used for bone resorption and bone formation are generally P1NP and CTX, which have been identified and recommended by international guidelines. These markers are regarded to reflect the bone mass change in the earlier stage of OP, but not for the diagnosis of osteoporosis. In this study, we found that P1NP and CTX were not sensitive enough to distinguish the OP group from the non-OP groups of both females and males (AUC < 0.7, *P >* 0.05, **Table S4**). ROC analysis showed that, in female subjects, the relative enrichment of amino sugar and nucleotide sugar metabolism, oxidative phosphorylation, as well as starch and sucrose metabolism could more efficiently distinguish the OP group from the non-OP groups (AUC > 0.7, *P* < 0.05, **Table S4**). For males, the richness of ABC transporters, amino sugar and nucleotide sugar metabolism, oxidative phosphorylation, purine metabolism, starch and sucrose metabolism, and the two-component system could be used as identification criteria (AUC > 0.7, *P* < 0.05, **Table S4**). The above findings indicated that the GM gene functional-based classifier could act as OP diagnostic markers and that the classifier model is different between female and male subjects.

### Network Analysis of the Relationship Among GM Composition, Gene Functional Signature, and BMD-Values/T-Score in Females and Males

**Figure 6:**
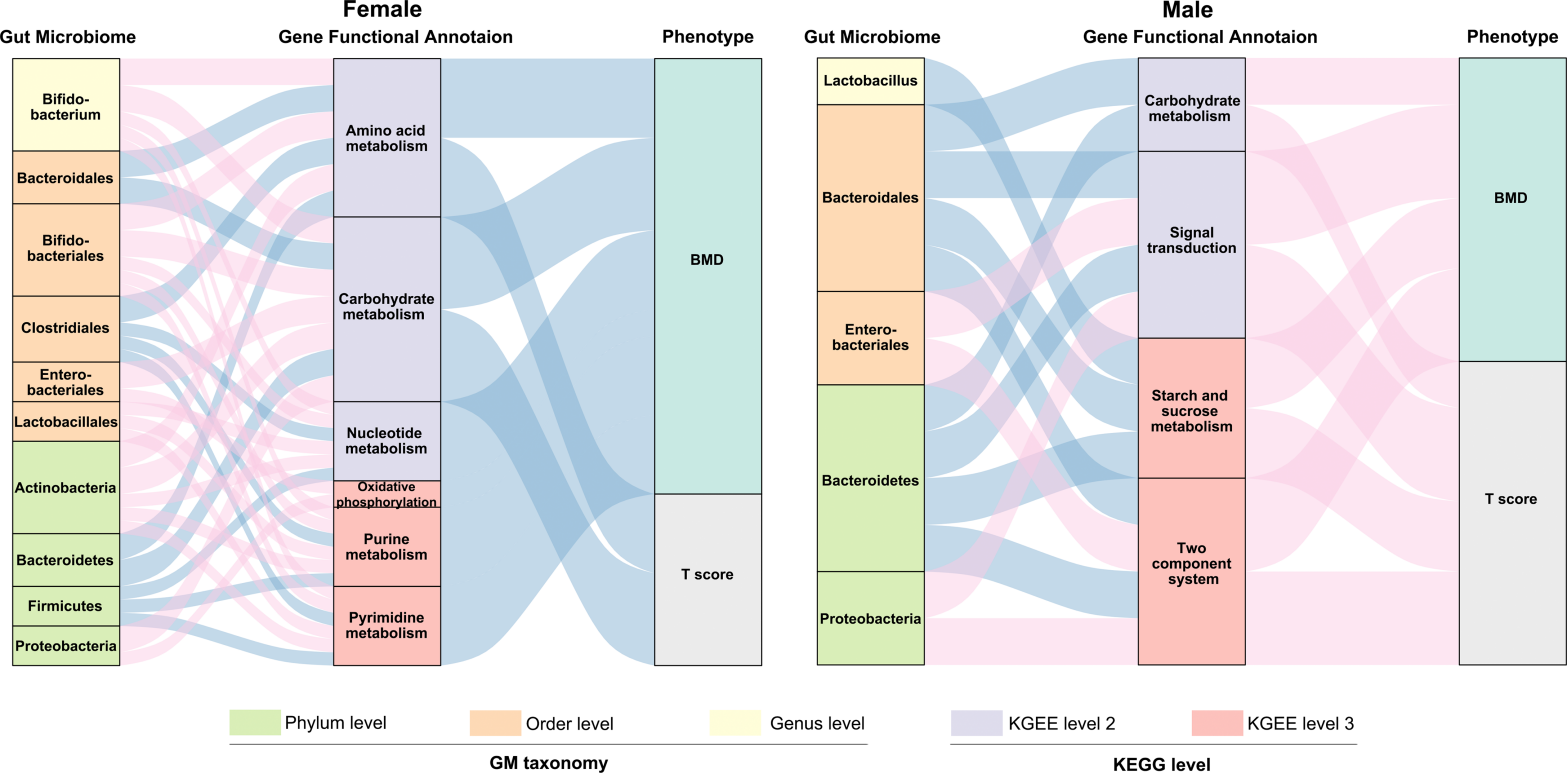
Interrelationship between GM composition, gene functional signature and BMD-value/T-score. Visualization of the correlation network according to Pearson correlation tests (*P* < 0.05 for all the correlations), red connection indicates a positive correlation, while blue connection represents a negative correlation. The detailed coefficient values were showed in **Table S5**.

We subsequently evaluated the correlation between the relative abundance of GM composition, gene functional annotation and BMD-values/T-score in both females and males (**Figure 6** and **Table S5**). We observed more correlation connections between the GM composition and gene functional signature in females than the males. For example, in the postmenopausal female participants, phyla *Firmicutes* and *Bacteroidetes* as well as order *Clostridiales* and *Bacteroidales* were negatively related with the several gene functional signature. The composition of phylum *Firmicutes* and order *Clostridiales* were relatively enriched in the females with normal-BMD values, which implied that *Firmicutes* and *Clostridiales* might play an important role in the maintenance of the normal bone physiological conditions by regulating the metabolic pathways. As mentioned previously, the GM composition was not significantly correlated with the BMD values in the males, while we found several connections of GM abundance and the gene functional annotations. The profiles within the gut microbiome and GM gene function allowed us to construct interaction networks for GM and GM-associated gene function modules, indicating that the gut microbiota may affect osteoporosis progression by interacting with host metabolism, and the connecting modules among the GM composition – gene functional signature – BMD/T score were different in the females and males.

## Discussion

The GM plays important roles in maintaining human health (Valdes et al., 2018). Various diseases and conditions could be affected by the GM imbalanced (Ho et al., 2015), including inflammatory disease, cardiovascular disease, diabetes, central nervous system diseases, and so on (Haran et al., 2019; Nemet et al., 2020; Pittayanon et al., 2020; Vangipurapu et al., 2020). As for bone health, the GM is a key regulator that affects postnatal skeletal development and skeletal involution (Zaiss et al., 2019). Although many studies have provided evidence that the GM may regulate bone metabolism, only a few studies have focused on patients with a low BMD (OP and ON) in the clinic. Recently, a research team from Wuhan, China, has reported on several taxa with an altered abundance and specific functional pathways in individuals with a low BMD (Li et al., 2019). Another study also has indicated that a reduced BMD is associated with taxon-specific signatures in the GM (Das et al., 2019). Previously, we also performed 16S rRNA sequencing to analyze the intestinal microbial diversity in a small sample size of OP patients and observed an inverse correlation between the number of bacterial taxa and BMD values (Wang et al., 2017). In the present research, we explored more deeply the influence of bacterial flora on bone metabolism by expanding the sample size and including metagenomic functional analysis. To the best of our knowledge, this study is the first to explore the association between the GM composition, as well as the GM gene functional signature and a reduced BMD in a human cohort, which included both female and male subjects suffering from OP and ON by combining 16S rRNA and metagenomic analysis.

In the first section, we found that a high prevalence of OP and ON occurred in postmenopausal women and males over 50 years old, and that the BMD loss in females was more than that in males of the same age. Also, significant vitamin D deficiency and insufficiency existed in all subjects. Consistently, the vitamin D levels were obviously lower in females than that in males, that women on average presented with vitamin D deficiency while the men presented with insufficiency, which could also reflect the sex-related distinctions between female and male. During the sample-collection process, we found that the prevalence of bone mass loss among older people was more severe than expected, which reminds us to pay greater attention to this “silent” disease than ever before. In fact, OP and the OP-related fractures remain largely under-diagnosed and under-managed in China (Yu and Xia, 2019), as well as in other countries (Wright et al., 2017; Cipriani et al., 2018). More specifically, we recruited participants and collected all samples from a small mountain village with a relatively poor socioeconomic status. Low income may be a significant factor related to the nutritional status and OP incidence (Park et al., 2016).

**Figure 7:**
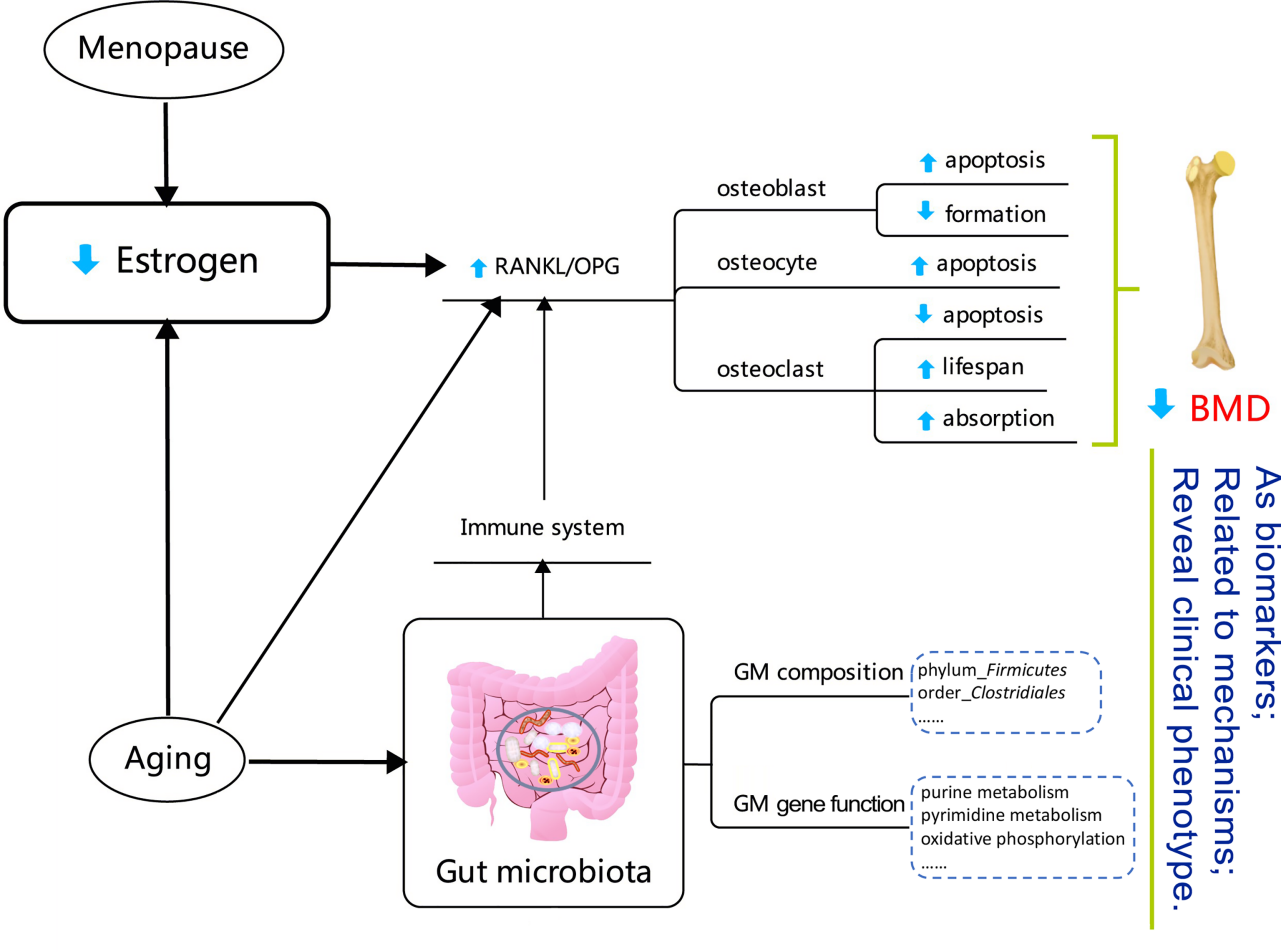
Diagram reflecting the correlations between gut microbiota and a low bone mineral density in the postmenopausal female. The detail explanation of the mechanisms is summarized in the *Discussion*. The content in the dotted box showed the BMD-related factors in the postmenopausal female participants in this study.

It has been reported that the GM composition depends on interactions between the host’s diet and gender within populations (Bolnick et al., 2014; Makki et al., 2018). Our finding that PCoA analysis of the OTU-level microbiota composition could be distinguished between females and males is also consistent with the relevant research (Bolnick et al., 2014). At the phylum level, the *Firmicutes* were enriched in both males and females. At the order level, the relative abundance of *Clostridiales* was positively correlated with the BMD values. Some of our results are consistent with recent related studies (Das et al., 2019; Li et al., 2019). Interestingly, the correlation tendency we observed above only existed in female subjects, although the GM composition was not very different between females and males at the phylum, order, and genus levels. The above results might reflect that, compared with what was observed in males, the GM is more sensitive to BMD alterations in females. This may be reasonable, since the sex-hormone drops dramatically in postmenopausal women. Estrogens and estrogen receptors (ERs) regulate metabolism in both normal physiology and in disease (Kulkoyluoglu-Cotul et al., 2019). More specifically, estrogen is an essential factor in bone metabolism and estrogen deficiency is the critical pathogenesis for the occurrence and development of PMOP (Li et al., 2020). Osteoclasts are a type of multinucleated cell that is formed by the fusion of precursor cells, and are critical for the skeletal development (growth and modeling), as they function in bone resorption. The key regulatory procedures associated with osteoclast production include the receptor activator of nuclear factor-κB (NF-κB) ligand (RANKL) binding to RANK on osteoclast precursor cells, which activates NF-κB and promotes osteoclast differentiation (Bartelt et al., 2018). Osteoblasts secret osteoprotegerin (osteoprotegerin, OPG), which also act as a soluble RANKL receptor, competitively binding RANKL and RANK, and thus inhibiting the formation of osteoclasts. The relative value of RANKL/OPG determines the degree of bone resorption and the procedure underlying bone metabolism. Menopause and aging are associated with a higher bone resorption/bone formation ratio (Bord et al., 2003; Berry et al., 2019). The function and adaptive capacity of the gastrointestinal tract declines with aging, and studies have demonstrated the various alterations in microbiota composition during the aging process (An et al., 2018). Besides, the host’s metabolism and immune status can be modulated by the GM (Sjogren et al., 2012; Bromberg et al., 2015; Ohlsson and Sjogren, 2015; Hernandez et al., 2016; Iqbal et al., 2016). The GM can also regulate bone mass by effecting on the immune system which, in turn, regulates osteoclastogenesis activity (Ohlsson and Sjogren, 2015). In addition, aging determines irreversible changes at the metabolic and physiological levels (Tesauro et al., 2017; Saferding et al., 2020). An estrogen deficiency may potentiate the adverse effects of aging, not only on bone, but also on lipid metabolism (Manolagas, 2010). In summary, alterations in the GM may act as biomarkers for the loss of BMD in PMOP, and may further reveal the clinical phenotype of the disease, as shown in **Figure 7**.

Our study performed in-depth metagenomic sequencing and bioinformatics analyses to determine the functional pathways and potential mechanisms of GM on BMD alterations. To improve the robustness of the study at a statistical level, we selected 57 out of the 113 fecal samples for further metagenomic analyses. After metagenomic sequencing, a slightly increased number of GM genes was observed in the female compared to the male subjects, which was similar to the 16S rRNA results, insofar as the females had a higher observed OTU than males. Overall, the most abundant genes were enriched at the metabolism level of the KEGG functional annotation. At the three levels of the KEGG functional profile, no significant difference was found between the female and male subjects (except that the relative abundance of signal transduction was decreased in males compared to that in females, with *P* < 0.05). Besides, as the enrichment of genes involved in several particularly functional pathways increased, the BMD values for female subjects tended to be lower, including carbohydrate metabolism, amino acid metabolism, and nucleotide metabolism in level 2, as well as purine metabolism, pyrimidine metabolism, and oxidative phosphorylation in level 3. On the contrary, the relative enrichment of genes annotated as carbohydrate metabolism and signal transduction in level 2 as well as the two-component system and starch and sucrose metabolism in level 3 were positively related to the BMD values in males. We also clarified that the two BTMs (CTX and P1NP) were not sensitive enough to distinguish the OP group from the non-OP groups (including both NC and ON groups). Instead, the functional-based classifiers of the GM gene performed efficiently when identifying the disease from the non-disease group, which indicates that alterations of an individual’s GM gene functional profile is sensitive to BMD changes to a certain extent, and that the GM and GM gene functional profile may provide some hints when monitoring disease progression in OP.

This study has some limitations. Firstly, the sex-based vitamin D deficiency and insufficiency detected within the current study may bias the findings, which need to be pay more attention. Besides, as the age-related changes and the dietary habit influent the composition of GM, it’s necessary to consider the confounders such as age, sex-hormones, diet or smoking habits of the individuals in larger cohort in the future study, to make the research more rigorous.

## Conclusion

Our study provides evidence in support of the notion that the composition of the GM is more sensitive to the BMD alterations in postmenopausal and older women. We have identified a number of novel relationships between gut microbes, GM gene functional signatures, and BMD values in both older females and males. Furthermore, the GM gene functional signatures exhibited potential performance in OP diagnosis. Taken together, our data provide significant biological findings related to the research of the GM and bone mineral metabolism. Besides, the extensive clinical information obtained for all participants is helpful for understanding the current status of OP incidence, especially in the elders. More functional studies are needed to explore the specific mechanisms underlying how the GM affects bone remodeling, which will be the focus of our future work.

## Data Availability Statement

The datasets presented in this study can be found in online repositories. The names of the repository/repositories and accession number(s) can be found below: https://www.ncbi.nlm.nih.gov/, PRJNA565497; https://www.ncbi.nlm.nih.gov/, PRJNA565546.

## Ethics Statement

This study was approved by the Biomedical Research Ethics Committee of Honghui Hospital, Xi’an Jiaotong University (No. 201801019), and was a part of the project “Diversity Analysis for Intestinal Flora in Patients with Primary Osteoporosis” registered at http://www.chictr.org.cn/index.aspx as #ChiCTR-1800019048#. The patients/participants provided their written informed consent to participate in this study.

## Author Contributions

JW and WG: project administration, funding acquisition, manuscript writing, supervision. YW, XG, and JL: data processing and analysis. YHZ and QL: cases inclusion and exclusion, blood sample collection and preparation. LW and YYZ: imaging examination. All authors contributed to the article and approved the submitted version.

## Funding

This study was supported by grants from National Natural Science Foundation of China (81702067); the Natural Science Foundation of Shaanxi Province (2020JM-692); Shaanxi Provincial Key Research and Development Program (2021SF-030); the Fundamental Research Funds for the Central Universities (20ykpy94, G2020KY0516); Yat-sen Scholarship for Young Scientist for WG, General Financial Grant from the China.

## Conflict of Interest

The authors declare that the research was conducted in the absence of any commercial or financial relationships that could be construed as a potential conflict of interest.

## Publisher’s Note

All claims expressed in this article are solely those of the authors and do not necessarily represent those of their affiliated organizations, or those of the publisher, the editors and the reviewers. Any product that may be evaluated in this article, or claim that may be made by its manufacturer, is not guaranteed or endorsed by the publisher.

## Supplementary Material

Supplementary Figure 1 Relative abundance of the main GM composition and their correlation with BMD values at different taxonomic levels in males. **(A)** Phylum level, **(B)** Order level, **(C)** Genus level.

Supplementary Figure 2 Observed gene numbers and their correlation with the BMD values in female and male group. **(A, B)** Observed gene numbers in female and male subjects, and in NC/ON/OP subgroups of female and male subjects. **(C, D)** Correlation analysis of gene numbers and BMD values in female and male subjects.

Supplementary Figure 3 Numbers of genes annotated at the six functional categories of the KEGG database. Numbers in each box show the number of genes.

Supplementary Figure 4 GM gene KEGG functional annotation for females and males. **(A)** Relative abundance of level 1 **(A)**, level 2 **(B)**, and level 3 **(C)** of KEGG annotations for females and males.

Supplementary Figure 5 Relative enrichment of candidate KEGG functional annotation in NC, ON and OP subgroups in females and males. **(A)** Relative enrichment of carbohydrate metabolism, amino acid metabolism and nucleotide metabolism in three subgroups of females. **(B)** Relative enrichment of carbohydrate metabolism and signal transduction in three subgroups of males. **(C)** Relative enrichment of purine metabolism, pyrimidine metabolism and oxidative phosphorylation in three subgroups of females. **(D)** Relative enrichment of two-component system, starch and sucrose metabolism in three subgroups of males.
