# Supplementary figures and images for: Gut Microbiome Signature Are Correlated With Bone Mineral Density Alterations in the Chinese Elders
Source: Front Cell Infect Microbiol. 2022 Mar 31;12:827575. doi: 10.3389/fcimb.2022.827575 (PMC9008261; doi:10.3389/fcimb.2022.827575)

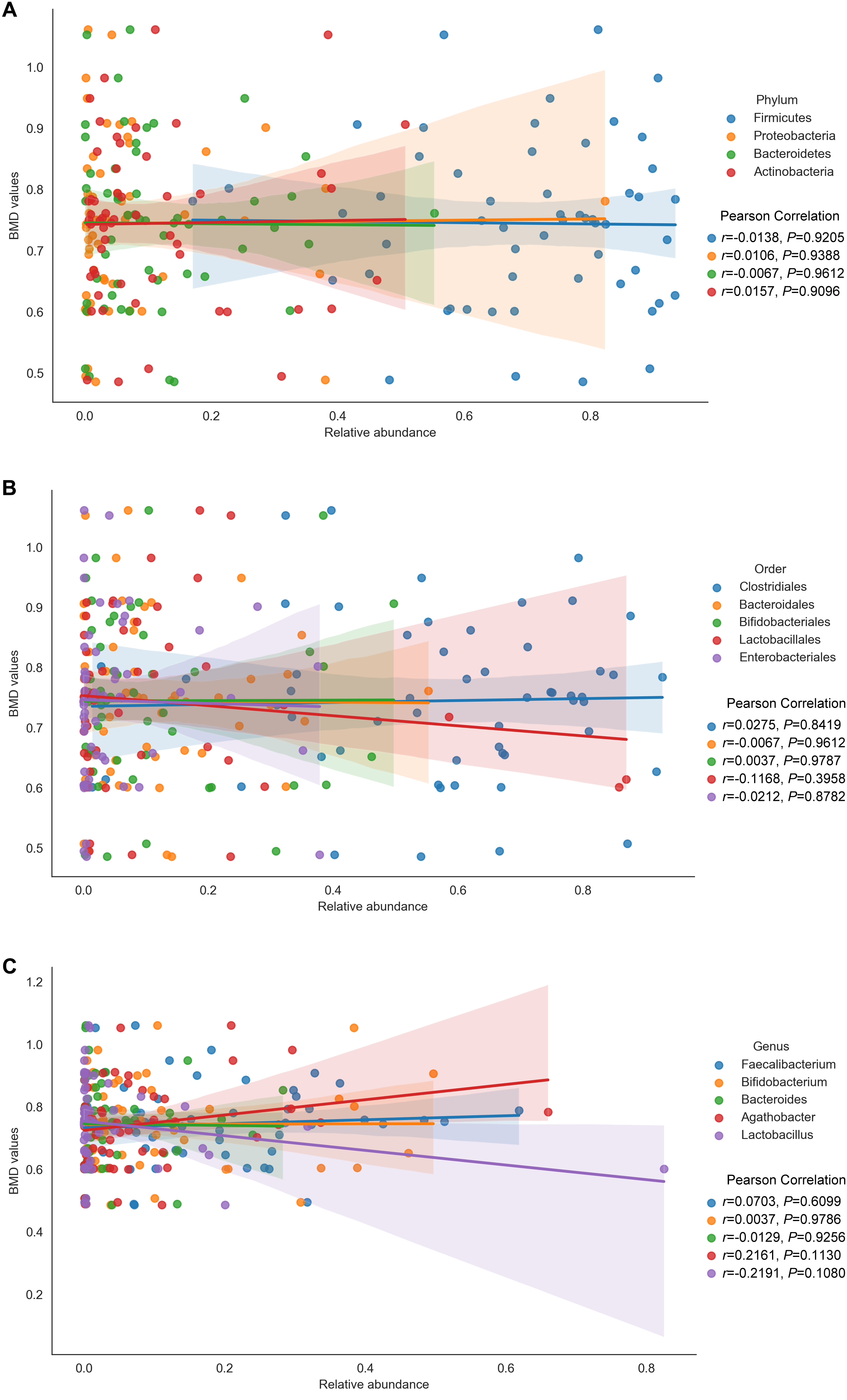

Supplement: Supplementary Figure 1 — Relative abundance of the main GM composition and their correlation with BMD values at different taxonomic levels in males. (A) Phylum level, (B) Order level, (C) Genus level. [file Image_1.tif]

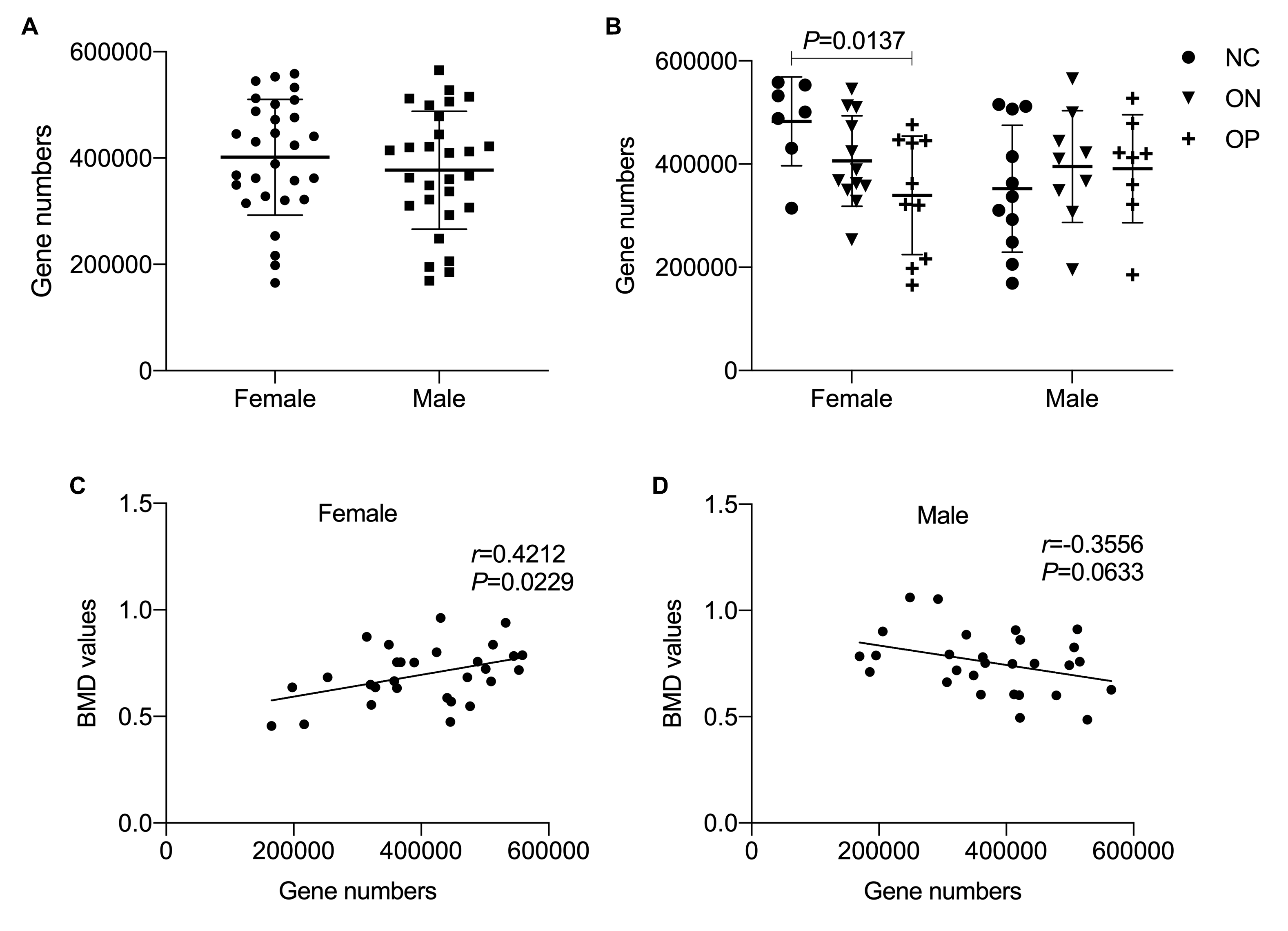

Supplement: Supplementary Figure 2 — Observed gene numbers and their correlation with the BMD values in female and male group. (A, B) Observed gene numbers in female and male subjects, and in NC/ON/OP subgroups of female and male subjects. (C, D) Correlation analysis of gene numbers and BMD values in female and male subjects. [file Image_2.tif]

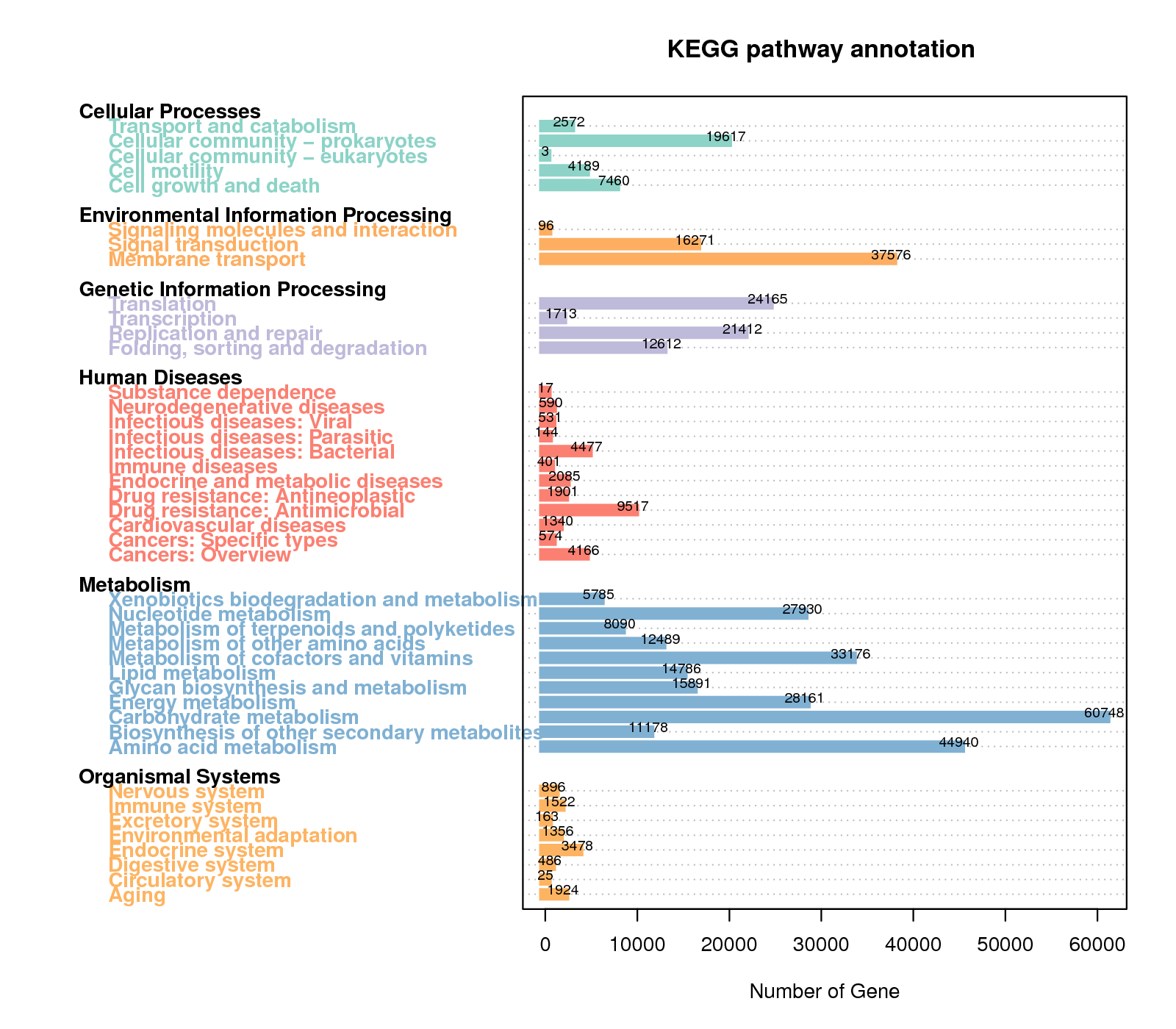

Supplement: Supplementary Figure 3 — Numbers of genes annotated at the six functional categories of the KEGG database. Numbers in each box show the number of genes. [file Image_3.tif]

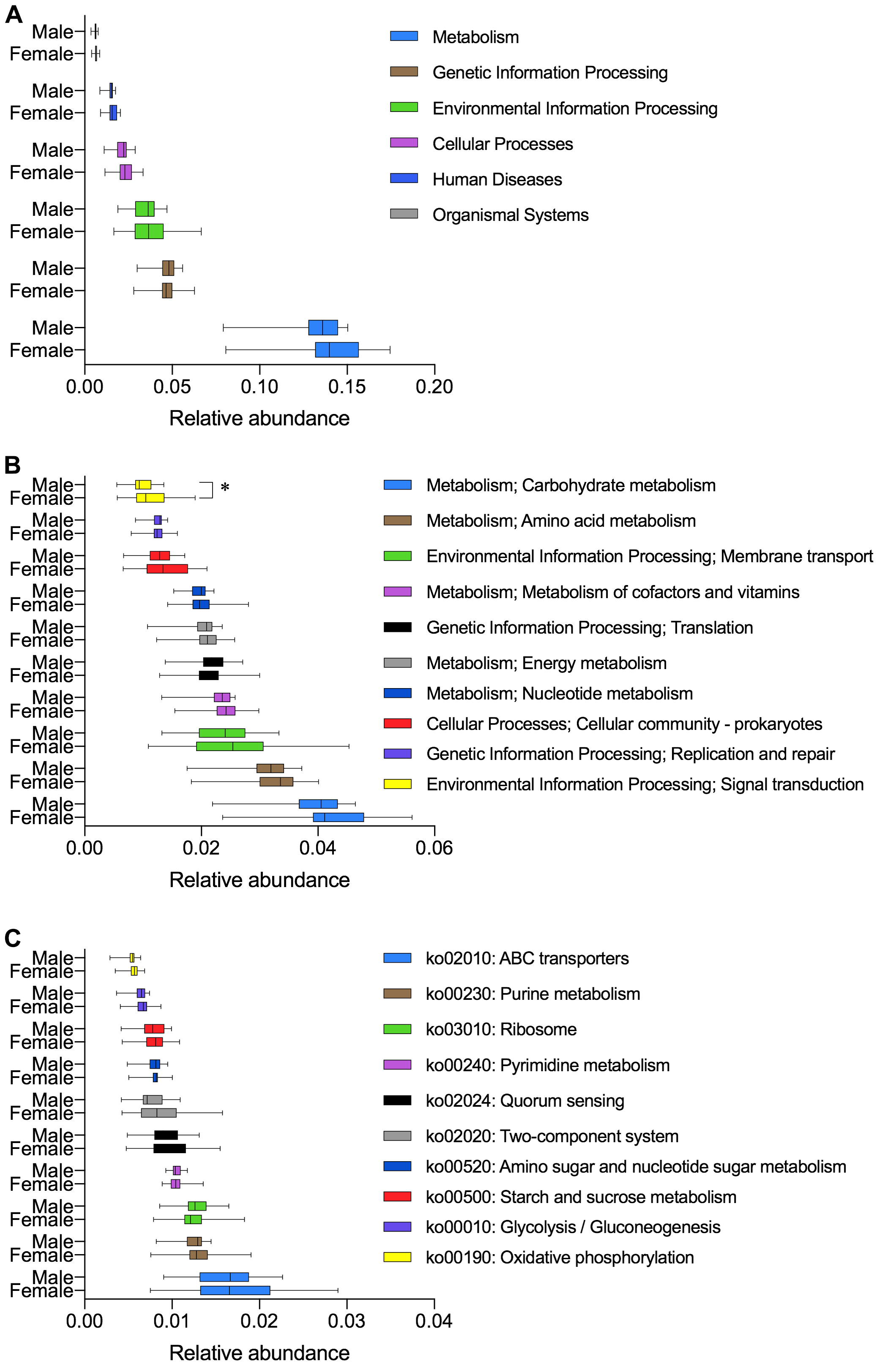

Supplement: Supplementary Figure 4 — GM gene KEGG functional annotation for females and males. (A) Relative abundance of level 1 (A), level 2 (B), and level 3 (C) of KEGG annotations for females and males. [file Image_4.tif]

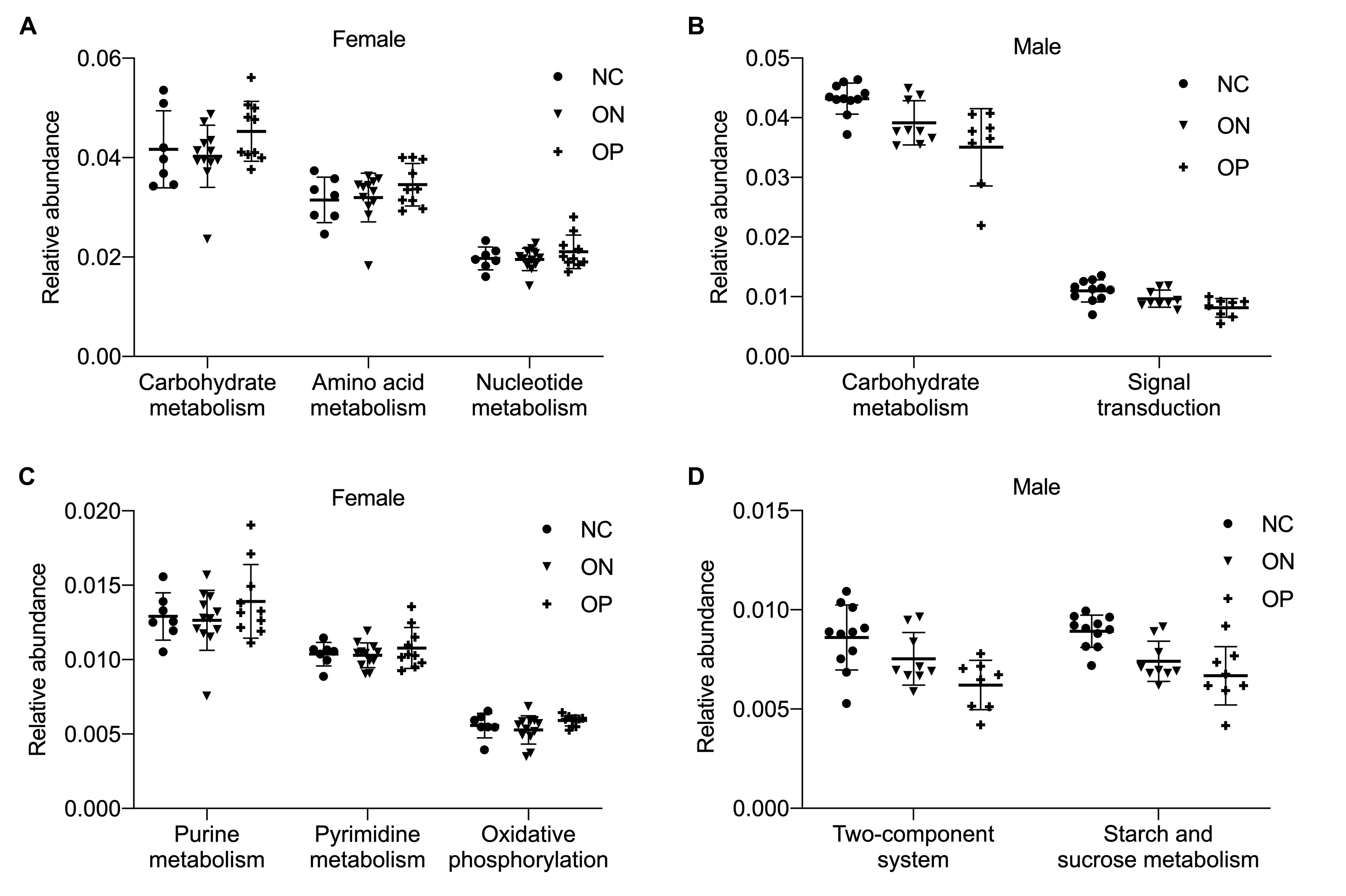

Supplement: Supplementary Figure 5 — Relative enrichment of candidate KEGG functional annotation in NC, ON and OP subgroups in females and males. (A) Relative enrichment of carbohydrate metabolism, amino acid metabolism and nucleotide metabolism in three subgroups of females. (B) Relative enrichment of carbohydrate metabolism and signal transduction in three subgroups of males. (C) Relative enrichment of purine metabolism, pyrimidine metabolism and oxidative phosphorylation in three subgroups of females. (D) Relative enrichment of two-component system, starch and sucrose metabolism in three subgroups of males. [file Image_5.tif]
