# Supplementary material for: Gut Microbiome Signature Are Correlated With Bone Mineral Density Alterations in the Chinese Elders
Source: Front Cell Infect Microbiol. 2022 Mar 31;12:827575. doi: 10.3389/fcimb.2022.827575 (PMC9008261; doi:10.3389/fcimb.2022.827575)
Supplement: Supplementary file 6 [file Table_1.docx]

**TABLE S1 |** Characteristics of the 113 subjects for whom 16S sequencing was performed

| Groups | | n | Age | M-age | BMI | Vitamin | ALP | CTX | P1NP | BMD | T score |
| --- | --- | --- | --- | --- | --- | --- | --- | --- | --- | --- | --- |
| Female | NC | 9 | 56.44±3.68 | 48.78±3.15 | 23.93±4.24 | 17.25±4.44 | 95.67±31.23 | 0.45±0.15 | 74.14±26.38 | 0.85±0.10 | -0.64±0.27 |
|  | ON | 28 | 56.54±6.17 | 47.11±1.83 | 23.89±3.17 | 18.07±5.10 | 100.96±26.23 | 0.42±0.14 | 74.99±25.44 | 0.71±0.08^#^ | -1.79±0.41^#^ |
|  | OP | 21 | 59.14±4.40 | 47.71±2.53 | 22.59±2.24 | 19.41±9.63 | 95.90±35.51 | 0.45±0.14 | 72.77±33.61 | 0.57±0.08^#△^ | -3.17±0.44^#△^ |
|  | Total | 58 | 57.47±5.32 | 47.59±2.36 | 23.43±3.07 | 18.43±6.95 | 98.31±30.20 | 0.43±0.14 | 74.05±28.31 | 0.68±0.13 | -2.11±0.98 |
| Male | NC | 19 | 58.42±6.18 |  | 24.05±2.97 | 24.62±10.56 | 89.95±26.52 | 0.33±0.14 | 53.16±15.34 | 0.87±0.09 | -0.37±0.65 |
|  | ON | 23 | 58.78±6.95 |  | 22.39±2.66 | 25.51±11.41 | 94.35±33.04 | 0.43±0.14^#^ | 67.83±23.79^#^ | 0.73±0.06^#^ | -1.74±0.41^#^ |
|  | OP | 13 | 60.31±5.66 |  | 22.67±3.26 | 21.26±7.29 | 94.85±41.38 | 0.43±0.23 | 62.51±38.24 | 0.59±0.08^#△^ | -3.02±0.62^#△^ |
|  | Total | 55 | 59.02±6.33 |  | 23.59±3.02 | 24.20±10.24^*^ | 92.95±32.63 | 0.39±0.17 | 61.50±25.99^*^ | 0.74±0.13^*^ | -1.57±1.15^*^ |

Compared with the female group, ^*^*P* < 0.05; compared with the NC subgroup in the same gender, ^#^*P* < 0.05; compared with the ON subgroup in the same gender, ^△^*P* < 0.05.
