# Supplementary material for: Gut Microbiome Signature Are Correlated With Bone Mineral Density Alterations in the Chinese Elders
Source: Front Cell Infect Microbiol. 2022 Mar 31;12:827575. doi: 10.3389/fcimb.2022.827575 (PMC9008261; doi:10.3389/fcimb.2022.827575)
Supplement: Supplementary file 7 [file Table_2.docx]

**TABLE S2 |** Characteristics of the 57 subjects for whom metagenome sequencing was performed

| Groups | | n | Age | M-age | BMI | Vitamin | ALP | CTX | P1NP | BMD | T score |
| --- | --- | --- | --- | --- | --- | --- | --- | --- | --- | --- | --- |
| Female | NC | 7 | 56.71±3.95 | 48.14±3.29 | 24.39±4.58 | 17.87±4.58 | 105.29±27.69 | 0.46±0.16 | 77.29±23.25 | 0.82±0.10 | -0.66±0.31 |
|  | ON | 12 | 54.42±2.75 | 47.17±2.21 | 22.77±3.40 | 19.02±3.46 | 99.25±25.9 | 0.38±0.13 | 64.88±21.38 | 0.74±0.07^#^ | -1.58±0.31^#^ |
|  | OP | 10 | 56.90±4.58 | 47.50±2.51 | 22.69±2.68 | 21.41±11.04 | 97.40±31.78 | 0.47±0.11 | 78.64±35.60 | 0.56±0.07^#△^ | -3.13±0.39^#△^ |
|  | Total | 29 | 55.83±3.81 | 47.52±2.53 | 23.13±3.44 | 19.56±7.10 | 100.07±27.61 | 0.43±0.13 | 72.62±27.33 | 0.70±0.13 | -1.89±1.04 |
| Male | NC | 11 | 56.91±5.41 |  | 23.89±2.95 | 25.08±9.01 | 83.73±26.02 | 0.33±0.14 | 51.57±15.56 | 0.88±0.10 | -0.29±0.76 |
|  | ON | 9 | 57.44±6.6 |  | 23.06±3.14 | 22.85±6.52 | 88.22±24.94 | 0.44±0.12^#^ | 70.71±16.78^#^ | 0.74±0.07^#^ | -1.63±0.49^#^ |
|  | OP | 8 | 61.00±4.72 |  | 22.14±2.74 | 21.32±6.42 | 103.38±47.39 | 0.41±0.11 | 59.29±38.92 | 0.60±0.08^#△^ | -2.98±0.58^#△^ |
|  | Total | 28 | 58.25±5.72 |  | 22.95±3.26 | 23.29±7.47 | 90.79±32.97 | 0.40±0.15 | 59.93±25.16 | 0.75±0.14 | -1.49±1.27 |

Compared with the NC subgroup in the same gender, ^#^*P*<0.05; compared with the ON subgroup in the same gender, ^△^*P* < 0.05.
