# Supplementary material for: Gut Microbiome Signature Are Correlated With Bone Mineral Density Alterations in the Chinese Elders
Source: Front Cell Infect Microbiol. 2022 Mar 31;12:827575. doi: 10.3389/fcimb.2022.827575 (PMC9008261; doi:10.3389/fcimb.2022.827575)
Supplement: Supplementary file 8 [file Table_3.docx]

**TABLE S3 |** Observed OTUs in different groups

| Groups | | n | 10 | 5278 | 11446 | 17164 | 22882 | 28600 | 34318 |
| --- | --- | --- | --- | --- | --- | --- | --- | --- | --- |
| Female | NC | 9 | 7.87±0.87 | 190.39±45.55 | 233.49±52.68 | 259.92±56.59 | 279.48±59.16 | 294.37±60.80 | 306.21±61.90 |
|  | ON | 28 | 7.50±0.80 | 182.87±36.36 | 225.33±41.18 | 252.57±42.36 | 271.93±44.34 | 287.35±45.69 | 300.10±47.23 |
|  | OP | 21 | 7.80±0.75 | 174.05±38.53 | 215.35±43.69 | 241.84±45.38 | 261.63±46.77 | 289.51±48.16 | 289.51±48.16 |
|  | Total | 58 | 7.67±0.80 | 180.84±38.37 | 222.98±43.64 | 249.82±45.45 | 269.37±47.24 | 284.73±48.51 | 297.22±49.47 |
| Male | NC | 19 | 7.08±1.37 | 165.73±38.88 | 202.65±45.60 | 226.07±49.00 | 242.13±51.35 | 254.98±52.41 | 265.42±53.89 |
|  | ON | 23 | 7.32±0.86 | 183.56±36.39 | 222.20±42.91 | 246.78±47.49 | 264.03±51.29 | 277.82±53.57 | 288.52±54.79 |
|  | OP | 13 | 6.77±1.54 | 162.45±63.65 | 202.90±74.02 | 225.58±77.84 | 243.03±81.99 | 256.37±84.39 | 267.77±86.95 |
|  | Total | 55 | 7.11±1.22^*^ | 172.41±45.12 | 210.89±52.48 | 234.61±56.34 | 251.50±59.66 | 264.86±61.57 | 275.64±63.19^*^ |

Compared with the female group, ^*^*P* < 0.05. The numbers in the header represents the sequences/sample of 16S sequencing.
