# Supplementary material for: Gut Microbiome Signature Are Correlated With Bone Mineral Density Alterations in the Chinese Elders
Source: Front Cell Infect Microbiol. 2022 Mar 31;12:827575. doi: 10.3389/fcimb.2022.827575 (PMC9008261; doi:10.3389/fcimb.2022.827575)
Supplement: Supplementary file 9 [file Table_4.docx]

**TABLE S4 |** Performance of criterions when distinguishing between OP and non-OP (ON and NC) in females and males

|  | T score, BTMs and KEGG annotation | Sensitivity | Specificity | AUC | P |
| --- | --- | --- | --- | --- | --- |
| Female  OP vs. non-OP | T score | 100.0 | 100.0 | **1.000** | **<0.001** |
|  | Amino sugar and nucleotide sugar metabolism | 90.0 | 73.7 | **0.795** | **0.001** |
|  | Oxidative phosphorylation | 70.0 | 84.2 | **0.737** | **0.016** |
|  | Starch and sucrose metabolism | 70.0 | 78.9 | **0.716** | **0.035** |
|  | P1NP | 70.0 | 63.2 | 0.579 | 0.533 |
|  | CTX | 70.0 | 73.7 | 0.676 | 0.104 |
| Male  OP vs. non-OP | T score | 100.0 | 100.0 | **1.000** | **<0.001** |
|  | ABC transporters | 62.5 | 80.0 | **0.744** | **0.014** |
|  | Amino sugar and nucleotide sugar metabolism | 100.0 | 60.0 | **0.794** | **0.001** |
|  | Oxidative phosphorylation | 87.5 | 75.0 | **0.813** | **0.003** |
|  | Purine metabolism | 87.5 | 55.0 | **0.719** | **0.048** |
|  | Starch and sucrose metabolism | 62.5 | 95.0 | **0.813** | **0.003** |
|  | Two component system | 100.0 | 55.0 | **0.813** | **<0.001** |
|  | P1NP | 37.5 | 95.0 | 0.581 | 0.583 |
|  | CTX | 75.0 | 55.0 | 0.563 | 0.578 |
