# Supplementary material for: Gut Microbiome Signature Are Correlated With Bone Mineral Density Alterations in the Chinese Elders
Source: Front Cell Infect Microbiol. 2022 Mar 31;12:827575. doi: 10.3389/fcimb.2022.827575 (PMC9008261; doi:10.3389/fcimb.2022.827575)
Supplement: Supplementary file 10 [file Table_5.docx]

**TABLE S5 |** Interrelationship between gut microbiota composition, GM gene functional signature and BMD value/T score in females and males

| Female | | | | | |
| --- | --- | --- | --- | --- | --- |
| Gut Microbiota | Gene Functional Annotation | Coefficient | Gene Functional Annotation | Phenotype | Coefficient |
| phylum_*Proteobacteria* | Oxidative phosphorylation | 0.5238 | Amino acid metabolism | BMD | -0.4214 |
| phylum_*Proteobacteria* | Carbohydrate metabolism | 0.4681 | Carbohydrate metabolism | BMD | -0.3841 |
| phylum_*Firmicutes* | Pyrimidine metabolism | -0.4755 | Nucleotide metabolism | BMD | -0.4202 |
| phylum_*Firmicutes* | Nucleotide metabolism | -0.4356 | Oxidative phosphorylation | BMD | -0.3687 |
| phylum_*Firmicutes* | Purine metabolism | -0.4025 | Purine metabolism | BMD | -0.4102 |
| phylum_*Bacteroidetes* | Carbohydrate metabolism | -0.4571 | Pyrimidine metabolism | BMD | -0.3898 |
| phylum_*Bacteroidetes* | Amino acid metabolism | -0.3911 | Amino acid metabolism | T | -0.3840 |
| phylum_*Actinobacteria* | Purine metabolism | 0.7026 | Carbohydrate metabolism | T | -0.3896 |
| phylum_*Actinobacteria* | Nucleotide metabolism | 0.6990 |  |  |  |
| phylum_*Actinobacteria* | Pyrimidine metabolism | 0.6364 |  |  |  |
| phylum_*Actinobacteria* | Amino acid metabolism | 0.5062 |  |  |  |
| phylum_*Actinobacteria* | Carbohydrate metabolism | 0.4244 |  |  |  |
| order_*Lactobacillales* | Pyrimidine metabolism | 0.4517 |  |  |  |
| order_*Lactobacillales* | Purine metabolism | 0.4346 |  |  |  |
| order_*Lactobacillales* | Nucleotide metabolism | 0.4179 |  |  |  |
| order_*Enterobacteriales* | Oxidative phosphorylation | 0.5293 |  |  |  |
| order_*Enterobacteriales* | Carbohydrate metabolism | 0.4710 |  |  |  |
| order_*Clostridiales* | Pyrimidine metabolism | -0.5490 |  |  |  |
| order_*Clostridiales* | Nucleotide metabolism | -0.5149 |  |  |  |
| order_*Clostridiales* | Purine metabolism | -0.4993 |  |  |  |
| order_*Clostridiales* | Amino acid metabolism | -0.3732 |  |  |  |
| order_*Bifidobacteriales* | Purine metabolism | 0.7003 |  |  |  |
| order_*Bifidobacteriales* | Nucleotide metabolism | 0.6955 |  |  |  |
| order_*Bifidobacteriales* | Pyrimidine metabolism | 0.6302 |  |  |  |
| order_*Bifidobacteriales* | Amino acid metabolism | 0.5090 |  |  |  |
| order_*Bifidobacteriales* | Carbohydrate metabolism | 0.4274 |  |  |  |
| order_*Bacteroidales* | Carbohydrate metabolism | -0.4572 |  |  |  |
| order_*Bacteroidales* | Amino acid metabolism | -0.3912 |  |  |  |
| genus_*Bifidobacterium* | Purine metabolism | 0.7003 |  |  |  |
| genus_*Bifidobacterium* | Nucleotide metabolism | 0.6955 |  |  |  |
| genus_*Bifidobacterium* | Pyrimidine metabolism | 0.6302 |  |  |  |
| genus_*Bifidobacterium* | Amino acid metabolism | 0.5090 |  |  |  |
| genus_*Bifidobacterium* | Carbohydrate metabolism | 0.4274 |  |  |  |
| Male | | | | | |
| Gut Microbiota | Gene Functional Annotation | Coefficient | Gene Functional Annotation | Phenotype | Coefficient |
| phylum_*Proteobacteria* | Two component system | 0.5615 | Carbohydrate metabolism | BMD | 0.4273 |
| phylum_*Proteobacteria* | Signal transduction | 0.5468 | Signal transduction | BMD | 0.3897 |
| phylum_*Bacteroidetes* | Starch and sucrose metabolism | -0.5818 | Starch and sucrose metabolism | BMD | 0.4182 |
| phylum_*Bacteroidetes* | Carbohydrate metabolism | -0.5359 | Two component system | BMD | 0.3957 |
| phylum_*Bacteroidetes* | Signal transduction | -0.3910 | Carbohydrate metabolism | T | 0.4999 |
| phylum_*Bacteroidetes* | Two component system | -0.3770 | Signal transduction | T | 0.4627 |
| order_*Enterobacteriales* | Two component system | 0.4001 | Starch and sucrose metabolism | T | 0.4625 |
| order_*Enterobacteriales* | Signal transduction | 0.3850 | Two component system | T | 0.4968 |
| order_*Bacteroidales* | Starch and sucrose metabolism | -0.5818 |  |  |  |
| order_*Bacteroidales* | Carbohydrate metabolism | -0.5359 |  |  |  |
| order_*Bacteroidales* | Signal transduction | -0.3910 |  |  |  |
| order_*Bacteroidales* | Two component system | -0.3770 |  |  |  |
| genus_*Lactobacillus* | Starch and sucrose metabolism | -0.4517 |  |  |  |
